# Development and Validation of Nomogram to Predict Acute Kidney Injury in Patients with Acute Myocardial Infarction Treated Invasively

**DOI:** 10.1038/s41598-018-28088-4

**Published:** 2018-06-27

**Authors:** Xuejun Zhou, Zhiqin Sun, Yi Zhuang, Jianguang Jiang, Nan Liu, Xuan Zang, Xin Chen, Haiyan Li, Haitao Cao, Ling Sun, Qingjie Wang

**Affiliations:** Department of Cardiology, The Affiliated Changzhou No.2 people’s Hospital of Nanjing Medical University, Changzhou, 213000 China

## Abstract

To identify patients who are likely to develop contrast-induced acute kidney injury (CI-AKI) in patients with acute myocardial infarction (AMI), a nomogram was developed in AMI patients. Totally 920 patients with AMI were enrolled in our study. The discrimination and calibration of the model were validated. External validations were also carried out in a cohort of 386 AMI patients. Our results showed in the 920 eligible AMI patients, 114 patients (21.3%) developed CI-AKI in the derivation group (n = 534), while in the validation set (n = 386), 50 patients (13%) developed CI-AKI. CI-AKI model included the following six predictors: hemoglobin, contrast volume >100 ml, hypotension before procedure, eGFR, log BNP, and age. The area under the curve (AUC) was 0.775 (95% confidence interval [CI]: 0.732–0.819) in the derivation group and 0.715 (95% CI: 0.631–0.799) in the validation group. The Hosmer-Lemeshow test has a p value of 0.557, which confirms the model’s goodness of fit. The AUC of the Mehran risk score was 0.556 (95% CI: 0.498–0.615) in the derivation group. The validated nomogram provided a useful predictive value for CI-AKI in patients with AMI.

## Introduction

The deterioration of renal function after contrast therapy is a well-known complication of invasive cardiovascular procedures. A large number of studies^1–4^ have shown that the development of contrast-induced acute kidney injury (CI-AKI) after coronary angiography (CAG) or percutaneous coronary intervention (PCI) is closely associated with increased rates of repeat revascularization, end-stage renal failure, myocardial infarction, early and late mortality in AMI.

To identify patients who are likely to develop CI-AKI after CAG or PCI is of great importance in prevention of contrast nephropathy. Several predictors are known to be associated with CI-AKI. Some existing scoring system had been developed, such as the Mehran risk score^5^, ACEF score^6^ and modified ACEF score^7^. For patients in the emergency room, however, these scores may not be as useful. Because some biomarkers are not routinely determined, and some of the patient’s full history are not known. In addition, CAG and left ventricular function have not been assessed. Therefore, it is necessary to take additional objective measures in the emergency room to improve identification of high-risk patients of CI-AKI.

The aim of this study was to provide a new tool to predict the risk of CI-AKI in patients with AMI undergoing CAG or PCI in order to decide the best preventive treatment option before procedure.

## Results

### Patient characteristics

Our study included 920 AMI patients in the department of Cardiology. The basic clinic characteristics for the training set (534 cases, mean age 68.3 ± 13.9, 69.5% male) and the validation set (386 cases, mean age 65.6 ± 13.3, 74.4% male) are listed in Table 1. In the training set, 114 patients (21.3%) developed CI-AKI, while in the validation set, 50 patients (13%) developed CI-AKI.

**Table 1:** Basic clinical and procedural characteristics.

Variables	Derivation cohort (n = 534)	Validation cohort (n = 386)
Age, years	68.3 ± 13.9	65.6 ± 13.3
Male, n%	371 (69.5%)	287 (74.4%)
SBP, mmHg	134.6 ± 26.3	124.3 ± 20.3
DBP, mmHg	78.2 ± 16.4	81.2 ± 18.3
Heart rate, bpm	82.3 ± 17.8	81.6 ± 16.5
Smoking	251 (47.0%)	209 (54.1%)
Alcohol intake	52 (9.7%)	59 (15.3%)
Hypertension	381 (71.3%)	220 (57.0%)
Diabetes	156 (29.2%)	88 (22.8%)
Serum creatinine, μmol/L	107.2 ± 66.7	78.4 ± 31.3
eGFR, mL/min/1.73 m^2^	61.6 ± 26.3	80.9 ± 26.7
HDL-C, mmol/L	1.23 ± 0.37	1.12 ± 0.38
LDL-C, mmol/L	2.41 ± 0.76	2.49 ± 0.86
Uric acid, μmol/L	350.1 ± 40.6	349.5 ± 84.2
Serum albumin, g/L	37.6 ± 1.6	38.1 ± 3.7
WBC, 10^9^/L	9.77 ± 3.86	9.29 ± 3.43
Neutrophil ratio(%)	77.8 ± 9.88	74.3 ± 13.1
Hemoglobin, g/L	129.2 ± 20.4	134.2 ± 19.5
LogBNP	3.19 ± 0.71	3.11 ± 0.68
Use of isotonic contrast agents	87 (16.3%)	76 (19.7%)
Hydration therapy	85 (15.9%)	85 (22.0%)
STEMI	465 (87.1%)	287 (74.4%)
PCI	427 (80.0%)	278 (72.0%)
CAG	107 (20.0%)	108 (28.0%)
Access site		
Radial access	524 (98.1%)	374 (96.9%)
Femoral access	10 (1.9%)	12 (3.1%)
Contrast volume		
>100 mL	25 (4.7%)	28 (7.3%)
≤100 mL	509 (95.3%)	358 (92.7%)
Hypotension before procedure		
Yes	14 (2.6%)	53 (13.7%)
No	520 (97.4%)	333 (86.3%)
CI-AKI		
Yes	114 (21.3%)	50 (13.0%)
No	420 (78.7%)	336 (87.0%)

CI-AKI = contrast-induced acute kidney injury, SBP = systolic blood pressure, DBP = diastolic blood pressure, eGFR = estimated glomerular filtration rate (mL/min/1.73 m^2^), HDL-C = High-density lipoprotein cholesterol, LDL-C = Low-density lipoprotein cholesterol, WBC = white blood cell, BNP = B-type natriuretic peptide, STEMI = ST segment elevation myocardial infarction, CAG = coronary angiography, PCI = percutaneous coronary intervention. Preoperational hypotension was defined as SBP lower than 90 mmHg before procedure.

### Predictors of CI-AKI

**Figure 1:**
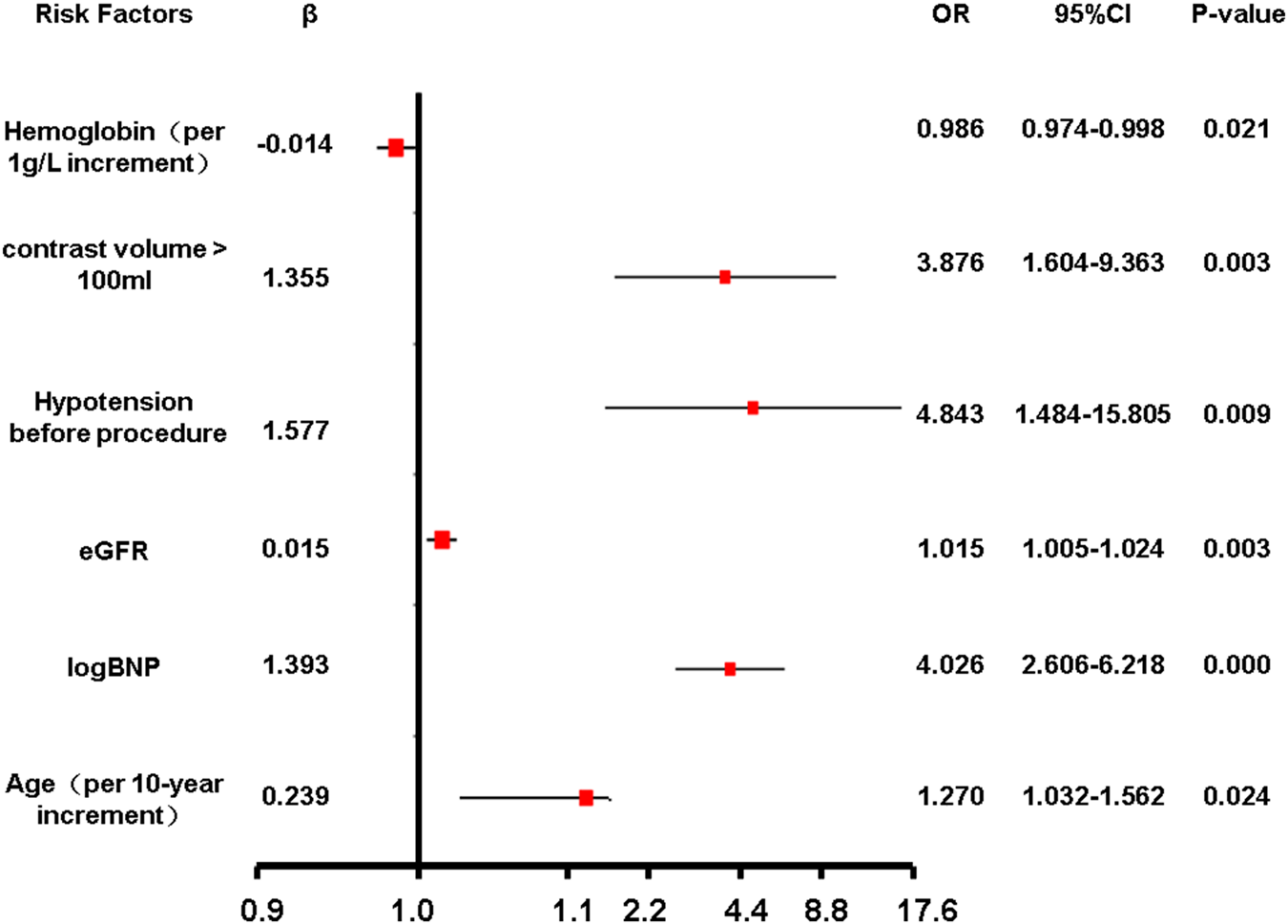
Independent risk factors of CI-AKI. Presented are multivariate logistic regression analysis to explore independent risk factors of CI-AKI in AMI patients. It indicated that hemoglobin, contrast volume >100 ml, hypotension before the procedure, eGFR, logBNP and age are independent risk factors of CI-AKI in AMI patients.

Details of the univariate comparison were showed in Table S1. Table S2 showed the multivariable logistic regression analyses in predicting CI-AKI in derivation cohort. For variables (p < 0.1) in Table S1, multivariable regression analysis was proceed in model 1. Considering the limitation of sample size and simplicity of the model, some variables of model 1 (0.05 < P < 0.1) were deleted in model 2. Finally, in order to simplify our model, Neutrophil ratio was deleted in model 3. The AUC of model 2 and 3 were 0.776 and 0.775, indicating that the predicting accuracy of the model 2 and 3 were the same as model 1 (Figure S1). Finally, Model 3 was chosen as our final model. It showed that hemoglobin, contrast volume >100 ml, hypotension before the procedure, eGFR, logBNP and age are independent risk factors of CI-AKI in AMI patients (Fig. 1). Since majority of the cases were performed radially (98.1% in derivation cohort and 96.9% in validation cohort), access site was not included as a covariate in the final model.

### Development of the nomogram

The probability of CI-AKI before CAG or PCI was assessed according to the multivariate logistic regression results. The equation was as follows: In (p/1−p) = 1.355*a + 1.577*b + 0.015*c + 1.393*d + 0.239*e − 0.014*f − 6.518. In the equation, p represents the probability of CI-AKI, a represents contrast volume >100 ml, b represents hypotension before procedure, c represents eGFR level, d represents logBNP level, e represents the age, and f represents hemoglobin level.

**Figure 2:**
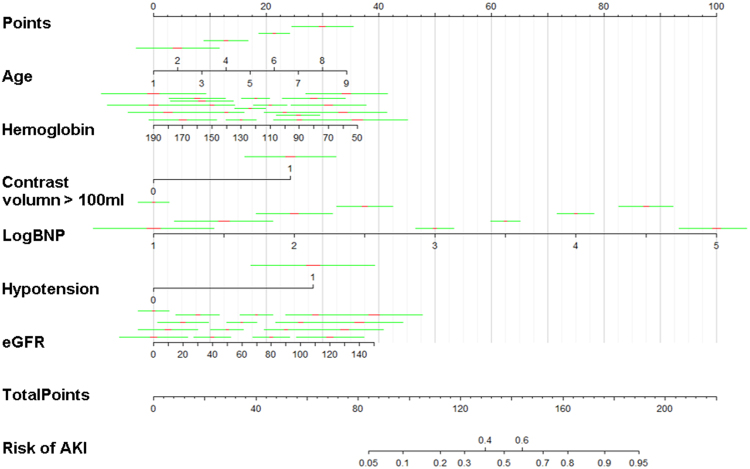
Nomogram of CI-AKI. First row: point assignment of the variables; second to seventh rows: predictors of CI-AKI; eighth row: total score of six predictors; ninth row: prediction of the risk of CI-AKI.

A nomogram (Fig. 2) presents our predictive model based on multivariate regression analysis model. There are nine rows in the nomogram: the first row was point assignment of the variables; second to seventh rows were predictors of CI-AKI; the eighth row was the total score of six predictors; and ninth row was the prediction of the risk of CI-AKI.

### Internal and external validation of the CI-AKI nomogram

**Figure 3:**
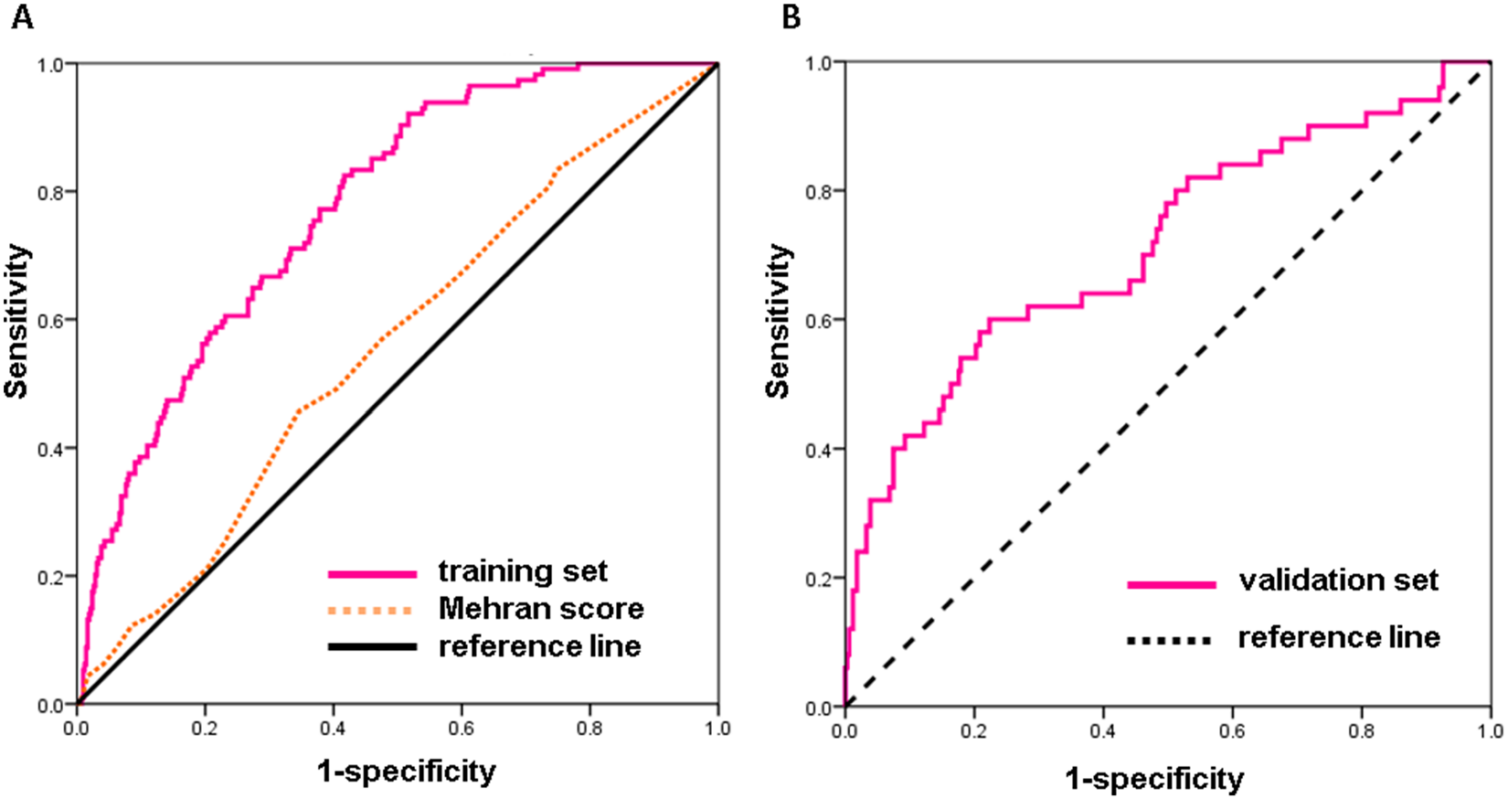
Validation of the nomogram and comparison with the Mehran risk score. (**A**) AUC of the training set: 0.775 (95% confidence interval [CI]: 0.732–0.819); AUC of The Mehran risk score: 0.556 (95% CI: 0.498–0.615). (**B**) AUC of validation set: 0.715 (95% CI: 0.631–0.799).

**Figure 4:**
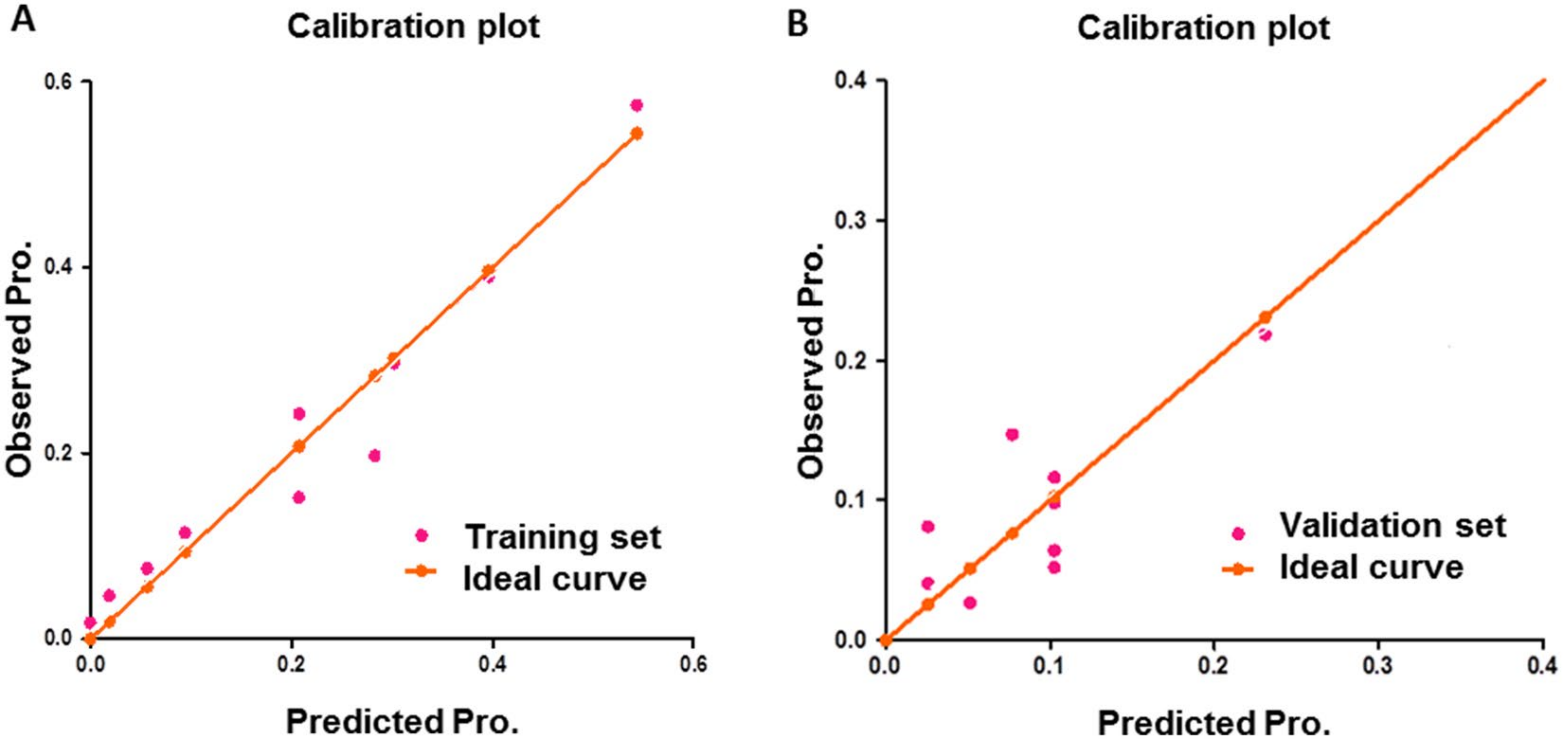
Calibration plots of the nomogram for the probability of CI-AKI. Calibration plots of training set (**A**) and validation set (**B**). The Hosmer-Lemeshow test had a p value of 0.557 in training set, while The Hosmer-Lemeshow test had a p value of 0.489 in the validation set.

Figure 3 showed the validation of the nomogram by the internal receiver operating characteristic (ROC) curves in the training set and external ROC in the validation set. The area under the curve (AUC) was 0.775 (95% confidence interval [CI]: 0.732–0.819) in the training set. The AUC were 0.715 (95% confidence interval [CI]: 0.631–0.799) in the validation set. Figure 4 showed the calibration plots of the nomogram for the probability of CI-AKI. The CI-AKI’s prediction probability of the model was consistent with the actual risk of CI-AKI. The Hosmer-Lemeshow test had a p value of 0.557 in training set and the Hosmer-Lemeshow test had a p value of 0.489 in validation set which confirms that the model fits nicely.

### Comparison of the nomogram with the Mehran risk score

Figure 3A also showed the AUC of the Mehran risk score was 0.556 (95% confidence interval [CI]: 0.498–0.615). It indicated that the new prediction model had a better performance in our studying population. Comparing probability of CI-AKI in our model (≥50%) with Mehran risk score (≥16) as arbitrary thresholds to define patients at low and high risk, the net reclassification improvement was 0.246 (NRI = 0.246, z = 3.732, P = 0.001). Discrimination improvement was confirmed by an integrated discrimination index (IDI) of 0.153 (z = 3.327, P = 0.001) when comparing with the Mehran risk score.

### Sensitivity analyses

We use original definition of AKI used in Mehran study, and then compare the AUC of the two. AUC of our nomogram model was 0.769, while AUC of Mehran risk score was 0.542. There were significant differences between the two, P < 0.001 (Figure S2). Furthermore, NRI was also calculated to compare potential clinical benefits achieved by new model with Mehran risk score. Using arbitrary thresholds of ≥50% for the current model and ≥16 for the Mehran risk score, the net reclassification index was 0.478 (NRI for non-events was 0.098, whereas NRI for events was 0.380). Discrimination improvement was confirmed by an integrated discrimination index (IDI) of 0.177 (95% CI: 0.140–0.213, P < 0.001) when comparing with the Mehran risk score. Obviously, our model still have a better performance in predicting AKI.

## Discussion

In this study, the main findings are as follows: (1) AMI patients undergoing CAG or PCI had a high incidence of postoperative CI-AKI (14.1%). (2) Hemoglobin, contrast volume >100 ml, hypotension before the procedure, eGFR, logBNP and age are independent predictors for CI-AKI in AMI patients treated invasively in our hospital. (3) Our model is feasible to predict CI-AKI in AMI patients in our hospital, and it is better to predict CI-AKI in AMI patients compared with the Mehran risk score.

CI-AKI is a frequent and serious postoperative complication of AMI patients undergoing CAG or PCI. In our study, the overall prevalence of CI-AKI in AMI patients is 17.8%, in which the incidence of the training set is 21.3%, and the incidence of the validation set is 13.0%, in line with the literature report^8,9^. Many factors are related to increased risk of CI-AKI. Reduced regional renal oxygen delivery cause inflammation, necrosis, and ischemia are common feature of CI-AKI^10^. Early development of contrast-induced acute kidney injury is considered as an independent predictor of in-hospital mortality in AMI patients treated invasively^11^. Thus we hope to develop a risk model to predict the risk of CI-AKI before procedure.

We consecutively selected and analyzed 920 AMI patients, among them 534 patients (training set) came from Center branch of our hospital, and the left 386 patients (validation set) came from Yanghu branch of our hospital, which located in 10 km away from center of the city. The CI-AKI risk model and nomogram were developed by the training set and were validated in the validation set. There are six predictors for CI-AKI in our model. Five variables, besides contrast agent volume, are available in the emergency room. HORIZONS-AMI study^12^ showed that age, hemoglobin, BNP, eGFR, heart rate, and LVEF were also independent predictors of CI-AKI. However, we did not find the heart rate as a risk factor for CI-AKI. It was also reported that in acute coronary patients (ACS) who underwent invasive management, radial access (RA) was associated with a reduced risk of AKI compared with femoral access (FA)^13^. However, in our study population, majority of our cases were performed radially. Thus, access site was not included as a covariate in the model.

High uric acid hematic disease, plasma albumin levels, intra-aortic balloon pump, number of stents, and hyperglycemia are not included in the final prediction model. Some of those factors have significant associations from the univariate analysis, the confounding factor disturbance must be removed before added into our model. In addition, we also hope that in the final prediction model of factors is relatively simple and easy for determination.

One strength of our study was that predictors of the model were routinely tested before CAG or PCI, which make it a reality for physician to calculate the total points and evaluate the risk of CI-AKI before the intervention and take further preoperative preventive methods to reduce the occurrence of CI-AKI. Another strength of our study was the training set and the validation set is from a different branch of our hospital. External validation of the discrimination and calibration of the model ensured our model of strong evidence to the CI-AKI events in our city. Furthermore, we compared the model with the Mehran risk score and it showed that our model had a better predictive value in AMI patients undergoing CAG or PCI in our region.

In recent years, there have been several prediction models of contrast induced CI-AKI in AMI patients. However, some of the risk model include too many risk factors during CAG or PCI, which makes it difficult to predict the occurrence of CI-AKI in the early stage^5–7^. The ACEF score includes LVEF value, which cannot be easily obtained in the emergency room^7^. The Mehran risk score^5^, which covers eight risk factors, is widely used for predicting the contrast induced nephropathy, but this score excludes patients with AMI and cardiogenic shock. Moreover, in our study, the Mehran risk score showed a lower predictive value to assess the CI-AKI risk of AMI patients than our model. Our study showed that the NRI was 0.246 when using arbitrary thresholds of ≥50% for the current model and ≥16 for the Mehran risk score. So in our model, we define thresholds of ≥50% as AKI high-risk groups. In order to minimize AKI occurrence, clinicians could calculate risk of AKI by nomogram, and take active preventive measures for patients over 50%.

There are some potential limitations of our study. Firstly, this study was only conducted in two branches of our hospital, so it was less methodological than studies using multicentric sampled populations. Secondly, variables during operation and novel biomarkers such as cycstatin C^14^, neutrophil gelatinase-associated lipocalin (NGAL)^15,16^, growth differentiation factor-15^17,18^, and so on, were not concluded in the model. It may improve the predictive value when combined these biomarkers. Thirdly, our model may not be extended to all kinds of AMI patients. To achieve a good model performance, we tried to expand the sample size and collected data from different intervention center. Further studies addressing these limitations are necessary so that this model could be improved and had a better predicting performance.

In summary, we developed a new CI-AKI risk model based mainly on preoperative risk factors and biomarkers and demonstrated that it provides an acceptable level of performance for predicting CI-AKI in a large cohort of AMI patients who underwent CAG or PCI. However, the applicability of this proposed risk model should be evaluated in additional studies.

## Methods

### Study populations

The derivation cohort was consisted of patients with AMI undergoing CAG or PCI in the department of cardiology in Central branch of our hospital. The validation cohort consisted of patients with AMI undergoing CAG or PCI in the department of cardiology in Yanghu branch of hospital (located in another district of the city, around 10 km away from the Central branch). The demographic and clinical characteristics of the inpatients between January 2013 and July 2017 were collected through electronic medical records retrospectively.

**Figure 5:**
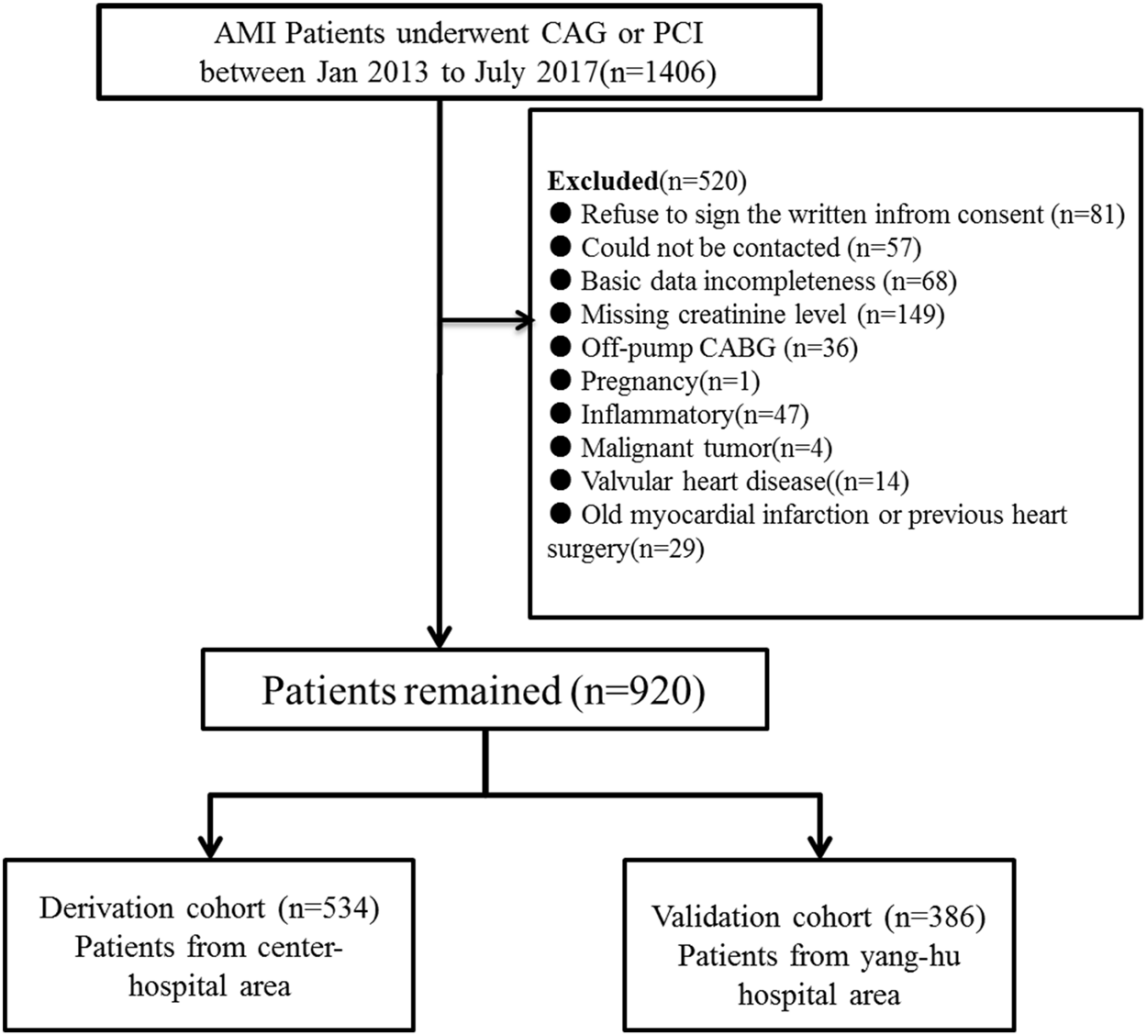
Study flow chart. AMI = acute myocardial infarction; CAG = coronary angiography; PCI = percutaneous coronary intervention.

All enrolled patients were more than twenty years old and were diagnosed with AMI. The definition of AMI was according to “the third universal definition of myocardial infarction from the Joint ESC/ACCF/AHA/WHF Task Force”^19^. All enrolled patients received CAG or PCI procedure. The exclusion criteria were as following: refuse to sign the written inform consent (n = 81), could not be contacted (n = 57), basic data incompleteness (n = 68), missing creatinine level (n = 149), off-pump CABG (n = 36), pregnancy (n = 1), Inflammatory (n = 47), malignant tumor (n = 4), valvular heart disease (n = 14), old myocardial infarction or previous heart surgery (n = 29). Our study chart flow was showed in Fig. 5.

### Ethical statements

Protocol approval was received from the institutional review board at our hospital, and each patient signed informed consent before participation. The study was approved by Changzhou No.2 People’s Hospital ethics committee. This Trial was registered in the Chinese clinical trials registry: ChiCTR1800014583. (http://www.chictr.org.cn/searchproj.aspx). In addition to this, we confirm that all methods were performed in accordance with the relevant guidelines and regulations.

### Collection of clinical and laboratory parameters

Basic clinic characteristics for the training set (n = 534) and the validation set (n = 386) were collected by reviewing the electronic medical records of the enrolled patients. The clinical variables such as age, gender, systolic blood pressure (SBP), diastolic blood pressure (DBP), body mass index (BMI), heart rate, and past history such as hypertension, diabetes mellitus were collected. Lifestyle factors such as history of cigarette smoking, alcohol consumption and use of medication were also collected. Preoperative hypotension was defined as SBP lower than 90 mmHg before procedure.

The blood samples of the hospitalized patients were collected and analyzed immediately at the time of admission. The following data were collected: white blood cell count (WBC), the ratio of neutrophils, hemoglobin, cardiac troponin I (cTnI), serum creatinine (Scr), uric acid, serum albumin, total cholesterol (TC), high density lipoprotein cholesterol (HDL-C), low density lipoprotein cholesterol (LDL-C), heamoglobina1c (HbA1c). Serum creatinine was also recaptured within 48 hours after CAG or PCI. All biochemical measurements were carried out in standard laboratory techniques. All the analyses were carried out by researchers who were blinded to the clinical data of the patients.

### Coronary angiography and interventional therapy

CAG or PCI by Seldinger technique via the radial artery puncture was conducted by experienced physicians in digital subtraction angiography (DSA) room. Femoral artery approach was adopted in patients who failed to do radial artery puncture. The procedural characteristics such as the contrast exposure time, volume of the contrast agent, the number of stents, use of isotonic contrast agent, hydration treatment were also collected.

### Definition of CI-AKI and eGFR

CI-AKI was defined as an absolute increase of serum creatinine of more than or equal to 0.3 mg/dL or increase to more than or equal to 150% from baseline 48-hour after CAG or PCI according to KDIGO criteria^20^. The estimated glomerular filtration rate (eGFR) was calculated by the abbreviated MDRD equation according to the baseline serum creatinine concentration^21^.

### Statistical Analysis

The demographic and clinical characteristics of patients in derivation and validation cohort were compared by Student t test or Wilcoxon rank sum test for continuous variables and χ^2^ or Fisher exact test for categorical variables. The main outcome of this study was the risk of CI-AKI on the basis of baseline characteristics. The logistic regression model was used to estimate the odds ratio (OR) and 95% CI of the risk of CI-AKI. Our study included the following predictors: age, hemoglobin, and contrast volume >100 ml, hypotension before the procedure, eGFR and logBNP. Nomogram was developed according to the logistic regression by the software R 3.2.3.

The c-index is the proportion of these predictions that is concordant. Values of the c-index range from 0.5 (no ability to discriminate) to 1.0 (full ability to discriminate). The c-index can be considered as a generalization of the area under the receiver operating characteristic (ROC) curve and is calculated by analyzing all possible patients. The calibration of the model was validated by hosmer-lemeshow test and calibration plots. External validations were also conducted in a cohort of 386 AMI patients in another hospital area.

Mehran risk score was also calculated for each patient in the training set. The two models are compared with the area under the ROC curve (AUROC). Moreover, net reclassification improvement (NRI) and the integrated discrimination Index (IDI) are also used to evaluate the improvement of the new risk model.

All analyses were performed using SPSS 22.0 (version 22.0, IBM Corp. Armonk, NY, USA) and R version 3.4.1 (the R Core Team; 2017 R; a programming environment for data analysis and graphic). All P are two sided. P value of less than 0.05 was considered statistically significant.

## Electronic supplementary material

Supplementary information
