# Supplementary material for: Development and Validation of Nomogram to Predict Acute Kidney Injury in Patients with Acute Myocardial Infarction Treated Invasively
Source: Sci Rep. 2018 Jun 27;8:9769. doi: 10.1038/s41598-018-28088-4 (PMC6021383; doi:10.1038/s41598-018-28088-4)
Supplement: Supplementary file 1 — Supplementary information [file 41598_2018_28088_MOESM1_ESM.pdf]

Development and Validation of Nomogram to Predict Acute Kidney Injury in Patients with Acute Myocardial Infarction Treated Invasively

Xuejun Zhou, Zhiqin Sun, Yi Zhuang, Jianguang Jiang, Nan Liu, Xuan Zang, Xin Chen, Haiyan Li, Haitao Cao, Ling Sun*, Qingjie Wang*

Department of Cardiology, The Affiliated Changzhou No.2 people's Hospital of Nanjing Medical University, Changzhou, China, 213000.

*Co-correspondence authors
Address for correspondence:
Ling Sun, Department of Cardiology, Affiliated Changzhou No.2 people's Hospital of Nanjing Medical University, 29 Xinglong Road, Changzhou, Changzhou 213000, China. Email: sunling85125@hotmail.com
Qingjie Wang, Department of Cardiology, Affiliated Changzhou No.2 people's Hospital of Nanjing Medical University, 188 Gehu Road, Changzhou, Changzhou 213000, China. Email: wang-qingjie@hotmail.com

Keywords: Acute Kidney Injury; Acute Myocardial Infarction; Nomogram;

***Table* S1.Basic clinical and procedural characteristics in derivation cohort**

**Variables**	**Non-AKI group (n=420)**	**AKI group (n=114)**	**P value**
Age, years	67.20±13.55	72.49±14.40	<0.001
Male, n%	307(73.1%)	64(56.1%)	0.001
SBP, mmHg	135.43±26.31	131.36±25.97	0.143
DBP, mmHg	78.90±15.89	75.70±17.86	0.064
Heart rate, bpm	81.45±16.45	85.22±21.93	0.045
Smoking	212(50.5%）	39(34.2%)	0.003
Alcohol intake	44(10.5%)	8(7.0%)	0.354
Hypertension	293(69.8%)	88(77.2%)	0.15
Diabetes	119(28.3%)	37(32.5%)	0.458
Serum creatinine, μmol/L	104.49±59.51	117.35±87.84	0.068
eGFR, mL/min/1.73m^2	61.65±24.44	61.42±32.37	0.935
HDL-C, mmol/L	1.23±0.38	1.21±0.32	0.704
LDL-C, mmol/L	2.42±0.72	2.40±0.87	0.883
Uric acid, μmol /L	351.11±41.58	346.71±36.90	0.306
Serum albumin, g/L	37.67±1.25	37.28±2.57	0.02
WBC, 10^9/L	9.64±3.86	10.26±3.82	0.124
Neutrophil ratio(%)	77.05±9.82	80.54±9.64	0.001
Hemoglobin, g/L	131.06±19.82	122.24±20.89	<0.001
LogBNP	3.07±0.73	3.61±0.44	<0.001
Use of isotonic contrast agents	65(15.5%)	22(19.3%)	0.403
Hydration therapy	63(15.0%)	22(19.3%)	0.333
STEMI	369(87.9%)	96(84.2%)	0.383
PCI or CAG			
PCI	342(81.4%)	85(74.6%)	0.136
CAG	78(18.6%)	29(25.4%)	
Contrast volume			
> 100mL	13(3.1%)	12(10.5%)	0.002
≤ 100mL	407(96.9%)	102(89.5%)	
Hypotension before procedure			
Yes	7(1.7%)	7(6.1%)	0.02
No	413(98.3%)	107(93.9%)	

AKI= acute kidney injury, SBP=systolic blood pressure, DBP=diastolic blood pressure, eGFR=estimated glomerular filtration rate (mL/min/1.73m^2), HDL-C=High-density lipoprotein cholesterol, LDL-C=Low-density lipoprotein cholesterol, WBC=white blood cell, BNP=B-type natriuretic peptide, STEMI=ST segment elevation myocardial infarction, PCI=percutaneous coronary intervention, CAG=coronary angiography, Preoperational hypotension was defined as SBP lower than 90mmHg before procedure.

Table S2. Multivariable logistic regression analyses in predicting CI-AKI in derivation cohort

	Model1			Model2			Model3		
	OR	95%CI	P-value	OR	95%CI	P-value	OR	95%CI	P-value
Age, per 10 years increasing	1.252	1.009	0.042	1.318	1.078-1.611	0.007	1.270	1.032-1.562	0.024
Male	0.729	0.390	0.321						
DBP	1.001	0.985	0.948						
Heart rate	1.000	0.986	0.965						
Smoking	0.968	0.521	0.918						
eGFR	1.016	1.007	0.001	1.013	1.004-1.023	0.006	1.015	1.005-1.024	0.003
Serum albumin	0.933	0.822	0.283						
Neutrophil ratio	1.037	1.010	0.007	1.036	1.011-1.061	0.004			
Hemoglobin	0.990	0.976	0.132	0.982	0.971-0.994	0.004	0.986	0.974-0.998	0.021
Hypotension before procedure	4.129	1.174	0.027	3.313	1.080-10.170	0.036	4.843	1.484-15.805	0.009
Contrast volume >100mL	3.386	1.376	0.008	3.462	1.466-8.174	0.005	3.876	1.604-9.363	0.003
LogBNP	3.871	2.467	0.000	1.084	0.758-1.549	0.660	4.026	2.606-6.218	<0.001
AUC	0.788			0.776			0.775		
P-value	reference			0.186			0.225		

Presented are multivariable logistic regression analyses. Three models are developed. AUC of the three models were compared.

***Figure S1*. AUC of the models from multivariable regression analysis.**

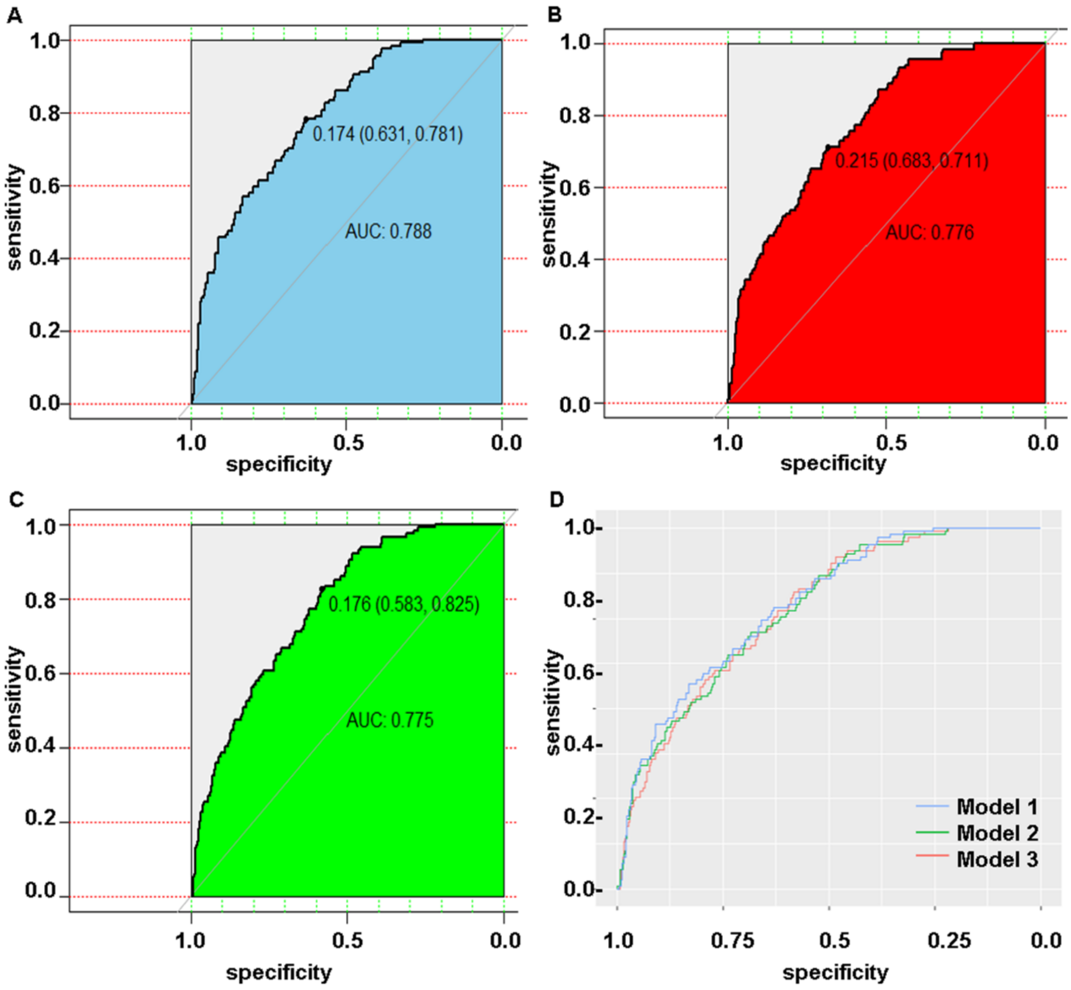

AUC of the models were showed. The AUC of model 2 (B) and 3 (C) were 0.776 and 0.775, indicating that the predicting accuracy of the model 2 and 3 were the same as model 1(A). D was the comparison of three models.

***Figure S2*. AUC of nomogram model and Mehran risk score by sensitivity analyses.**

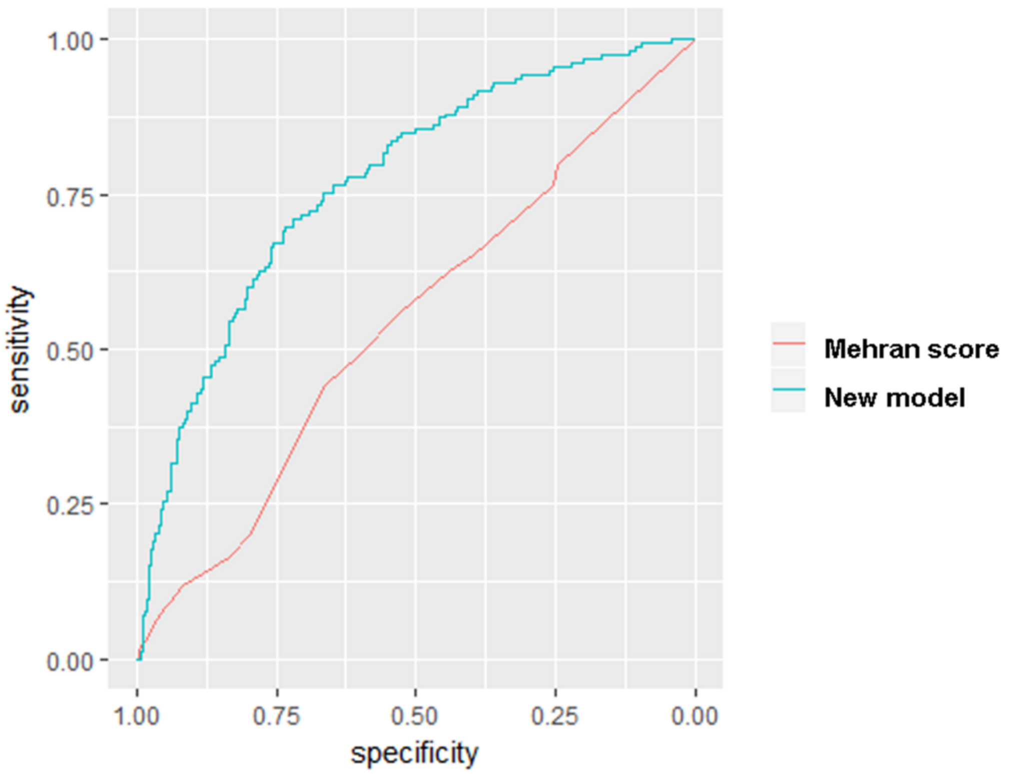

AUC of our model was 0.769, while AUC of Mehran risk score was 0.542, indicating that there were still significant differences between the two model using the original definition of AKI used in the Mehran study ($P < 0.001$).
